# Evaluating Patient Experience With Genomic Medicine: A Content Analysis of National Cancer Institute–Designated Cancer Centers’ Websites

**DOI:** 10.2196/76549

**Published:** 2025-10-21

**Authors:** Amanda Sara Andriessen, Loren Saulsberry

**Affiliations:** 1 Center for Personalized Therapeutics, University of Chicago, Chicago, IL, United States; 2 Department of Public Health Sciences, University of Chicago, MC 2000, 5841 S Maryland Avenue, Chicago, IL, 60637, United States, 1 773-702-2543; 3 Department of Medicine, Section of Hematology/Oncology, The University of Chicago, Chicago, IL, United States

**Keywords:** genetic testing, cancer centers, personalized medicine, cancer communication, patient experience, content analysis

## Abstract

**Background:**

National Cancer Institute–designated cancer centers (NCI-CCs) throughout the United States are mandated to translate state-of-the-art cancer research to communities and enhance clinical care for patients within their catchment areas. NCI-CCs play a vital role in national cancer initiatives focused on optimizing cancer care via personalized medicine in which improved risk assessment, screening, and genetic testing are foundational. In this era of targeted personalized care, although genetics has been incorporated into cancer centers, it is unknown how these innovations are being communicated to the public and communities served on cancer center websites. There is particularly limited knowledge surrounding how NCI-CCs publicly communicate their efforts to integrate patient-reported experiences with genomics to fulfill their overall mission and reduce the cancer burden in their catchment areas.

**Objective:**

The objective of this study was to evaluate how NCI-CCs publicly share information on their websites related to cancer center programming and activities to measure and incorporate patients’ experiences with the use of genetics to guide cancer care.

**Methods:**

For all NCI-CCs providing clinical care (N=65), we conducted a review of publicly available and published information and assessed five domains relevant to patients’ experiences with genomic medicine: whether NCI-CCs (1) provided genetic testing, (2) directly expressed a goal of delivering personalized care, (3) provided pharmacogenomic testing, (4) assessed patient-reported experience measures with genomic medicine (including patient-reported outcomes [PROs] and other patient experience measures [OPEMs]), and (5) indicated an established infrastructure or set of resources to evaluate patient experience. We conducted a content analysis of the publicly available websites of NCI-CCs using the validated directed approach to content analysis. We quantified the results of our content analysis using count measures based on a binary (yes or no) coding scheme.

**Results:**

While almost all the NCI-CCs (64/65, 98%) discussed providing personalized care and performing genetic testing on their websites, we found that 58% (38/65) indicated online that they assessed PROs or other patient experience measures with genomic medicine. Fewer centers (25/65, 38%) discussed on their websites having a mechanism for evaluating patients’ experiences with genomic medicine that captured broader types of information beyond PROs, such as measures of patient education or care team communication. Finally, approximately 1 in 3 NCI-CCs (23/65, 35%) indicated having an established infrastructure with departmental resources dedicated to monitoring patients’ experiences. These centers reflecting a built-in infrastructure were 8% to 12% more likely to publicly communicate targeted activities to assess patients’ experiences with genomic medicine.

**Conclusions:**

With the burgeoning use of genomics in research and clinical care, comprehensive evaluation and incorporation of measures of patients’ experiences with genomic medicine present a key opportunity to enhance cancer care at NCI-CCs.

## Introduction

### The National Cancer Institute’s Cancer Centers Program

Established by the National Cancer Act in 1971, the National Cancer Institute’s (NCI) Cancer Centers Program has worked for over 50 years to support and unify cancer research efforts and clinical care across institutes nationwide [1]. Achieving the status of an NCI-designated comprehensive cancer center (NCI-CC) is the crowning achievement for US cancer-focused research centers and hospitals. As NCI-CCs, these centers shape the future of cancer care through supporting the translation of laboratory discoveries to clinic, community-focused programming, and developing advancements in the prevention, diagnosis, and treatment of cancer for their catchment areas [2,3].

NCI-CCs play a vital role in national cancer initiatives focused on optimizing cancer care via personalized medicine in which improved risk assessment, screening, and genetic testing are foundational [4,5]. Personalized medicine is a burgeoning field focused on providing patient-specific treatment by considering unique factors that can impact individuals’ health, most principally their genetic makeup [6,7]. Personalized medicine in oncology includes several types of care, including genetic testing (1) for germline mutations related to inherited susceptibility to cancer, (2) for somatic mutations providing tumor characterization, and (3) for pharmacogenomics (PGx) profiles that predict responses to medications (eg, potential efficacy and side effects).

### Patient-Provider Communication Regarding Genetic Testing

Efforts to understand the human genome in the last 30 years have changed the way in which we look at and treat cancer [8-10]. Despite these advancements and best practice recommendations, genetic testing is only performed for a fraction of eligible patients. In an observational study of the prevalence of germline genetic testing following a cancer diagnosis, only 6.8% of patients diagnosed between 2013 and 2019 underwent germline genetic testing [11]. While up to 15% of cancer cases are attributable to inherited mutations, over 70% of patients with a breast or ovarian cancer diagnosis reported not being advised on any genetic testing by their providers [12]. Moreover, these germline genetic testing rates are lower among Asian, Black, and Hispanic patients in comparison to non-Hispanic White patients with comparable cancers [12-16]. In a systematic review and meta-analysis of patients with ovarian cancer, 40% of White patients completed genetic testing, whereas only 26% and 14% of Black and Asian patients, respectively, underwent such testing following a diagnosis [17].

Among patients newly diagnosed with breast cancer and high risk of genetic mutations, the number 1 reason cited for not receiving germline genetic testing was not expense or lack of interest but rather the lack of a physician’s recommendation for testing [13]. Similarly, health care professionals identify themselves as the responsible party for identifying individuals who would benefit from genetic testing and disclosing the results to patients. Despite this recognition, communication barriers between providers and patients regarding genetic testing and results continue to contribute to low uptake among eligible patients [1,18,19].

To ensure that all patients are able to fully benefit from genetic advancements in cancer care, we need to address communication regarding genetic testing for both cancer screening and treatment at cancer centers. While many national and international organizations have published guidelines for genetic testing to address this testing gap, effective communication and outreach regarding cancer genetic testing remains an active area of study and development [8]. Even in cases where patients have received testing, they often cite misunderstanding the risk implications of such testing for them and the utility of results to their family members [20].

### Patients’ Experiences With Genetic Testing

One established way to address these communication gaps and understand the underlying cause for testing disparities is to evaluate and understand patients’ experiences within the health care system. The National Academy of Medicine identifies a shortage of patient experience evaluations as a contributing factor to the quality of personalized patient care [21]. Supporting patient experience is fundamental to improving health care across many domains through improving patient engagement, transparency, and patient-provider communication [22]. Previous studies have addressed the implementation of such genetic testing to guide treatment and physicians’ perspectives on its utility, yet they often fall short of considering patient experiences. However, when patient experience evaluation is incorporated in cancer care, patients and their families benefit from a better understanding and risk communication regarding their genetic test results [23-25]. Understanding patient perspectives and the accessibility of genetic testing in cancer care is a key part of equitably implementing precision oncology care [26,27].

Evaluating patient engagement and communication in the delivery of personalized medicine requires measurable responses and feedback from the patients themselves. Patient-reported experience measures (PREMs), such as patient satisfaction scales and knowledge assessments, are vital to providing comprehensive patient-centered care [28,29]. We define PREMs to include all measures that encapsulate a patient’s experience with the health care system in line with the US Agency for Healthcare Research and Quality [28]. PREMs have been associated with clinically-relevant differences in patient understanding, trends in emergency room visits, and patterns of overall survival [30-33]. A subset of PREMs, patient-reported outcomes (PROs), includes measures such as quality of life assessments and symptom reporting in oncology practice to facilitate communication between physicians and patients regarding clinical care [34,35]. Moreover, other patient experience measures (OPEMs) can capture alternative aspects important to personalizing care, such as the way in which patients view their illness and interact with their care team.

At the individual level, PREMs can facilitate patient-provider communication and improve symptom management, and on a public and administrative level, PREMs can inform the implementation of burgeoning genetic technologies into equitable cancer care [31,36]. As leaders, and in alignment with NCI priorities, NCI-CCs’ incorporation of PREMs into patient care and research efforts, especially alongside the implementation of genomic medicine, may facilitate improving care quality and addressing cancer health disparities.

Outside of the examination room, the internet, and specifically patient-facing information on cancer center websites, serves as a valuable resource for patients to become informed about their cancer care and the resources available to them that they can pursue or advocate for [37]. In these cases, online information is crucial to communication with patients and health education beyond the limited time frame of the health encounter. Cancer center websites can bolster communication efforts with the community, provide information transparency, and promote resources to patients [38]. It is important that these websites disseminate information about genetic testing resources available to their catchment area and their efforts to incorporate patients’ experiences. Furthermore, cancer center websites may reflect certain priorities and efforts with regard to genetic testing programs, such as expanding screening for hereditary risk, personalized medicine, and patient outreach. Few studies have evaluated the content of the information that cancer centers directly communicate to the public [39,40], and none of these studies has specifically addressed patients’ experiences with genomic medicine. One study evaluated cancer center websites for information about a novel oncology intervention [39], whereas another assessed the content of cancer center public advertising [40]. Limited research exists on the efforts of NCI-CCs to incorporate patient experience with genomic medicine implementation, let alone their public communication of these efforts. Therefore, to further characterize the integration and accessibility of PREMs and genetic testing within NCI-CCs, we reviewed publicly available content to determine their communication about PREMs in their patient care and research efforts related to the implementation of genomic medicine.

## Methods

### Study Inclusion Criteria

A list of 72 US NCI-CCs was originally compiled from the National Institutes of Health NCI’s list of designated cancer centers in October 2023 [41]. The NCI Cancer Centers Program encompasses 3 subdesignations: basic cancer centers, clinical cancer centers, and comprehensive cancer centers [3]. To be evaluated, the centers had to provide direct clinical care; therefore, of the 72 NCI-CCs, 7 basic laboratory cancer centers were excluded from our content analysis, leaving 65 evaluable centers.

### Content Analysis

The websites of these centers are critical access points for the public, communicating the centers’ goals and accomplishments along with providing a digital footprint of their community-serving and research activities [42,43]. Thus, we conducted a content analysis of the publicly available websites of NCI-CCs using the validated directed approach to content analysis previously described in the literature [44]. The findings of our content analysis were quantified using count measures based on a binary (yes or no) coding scheme. When a specific content element as outlined by our domain criteria was discovered on a cancer center website, its presence was recorded using “yes” in the study database. To arrive at a quantitative count measure, at least one “yes” in a domain would correspond to counting that cancer center as publicly communicating about that particular domain.

### Website Review Process and Variable Coding

Each website assessment started with the NCI-CC website home page [41], which directs the user through designated links to each specific cancer center’s website. Once navigated to the specific cancer center’s website, the reviewer first assessed that website’s directory (eg, main tabs or prominent links to web pages within the site that referenced domain criteria). Second, the reviewer systematically followed the links of all matches to domain criteria to their resulting web pages; this included a systematic review of the website’s directory as well as searches based on a specified term list on the main website’s internal search bar. Following these procedures, when the reviewer observed domain criteria, the presence of that specific content element was recorded in the study database using a binary indicator (“yes” for present or “no” for absent). At least one instance in which that domain criterion was discussed was required for recording that the NCI-CC publicly communicated regarding that specific domain on their website.

### Domain Criteria for Data Collection

**Figure 1.**
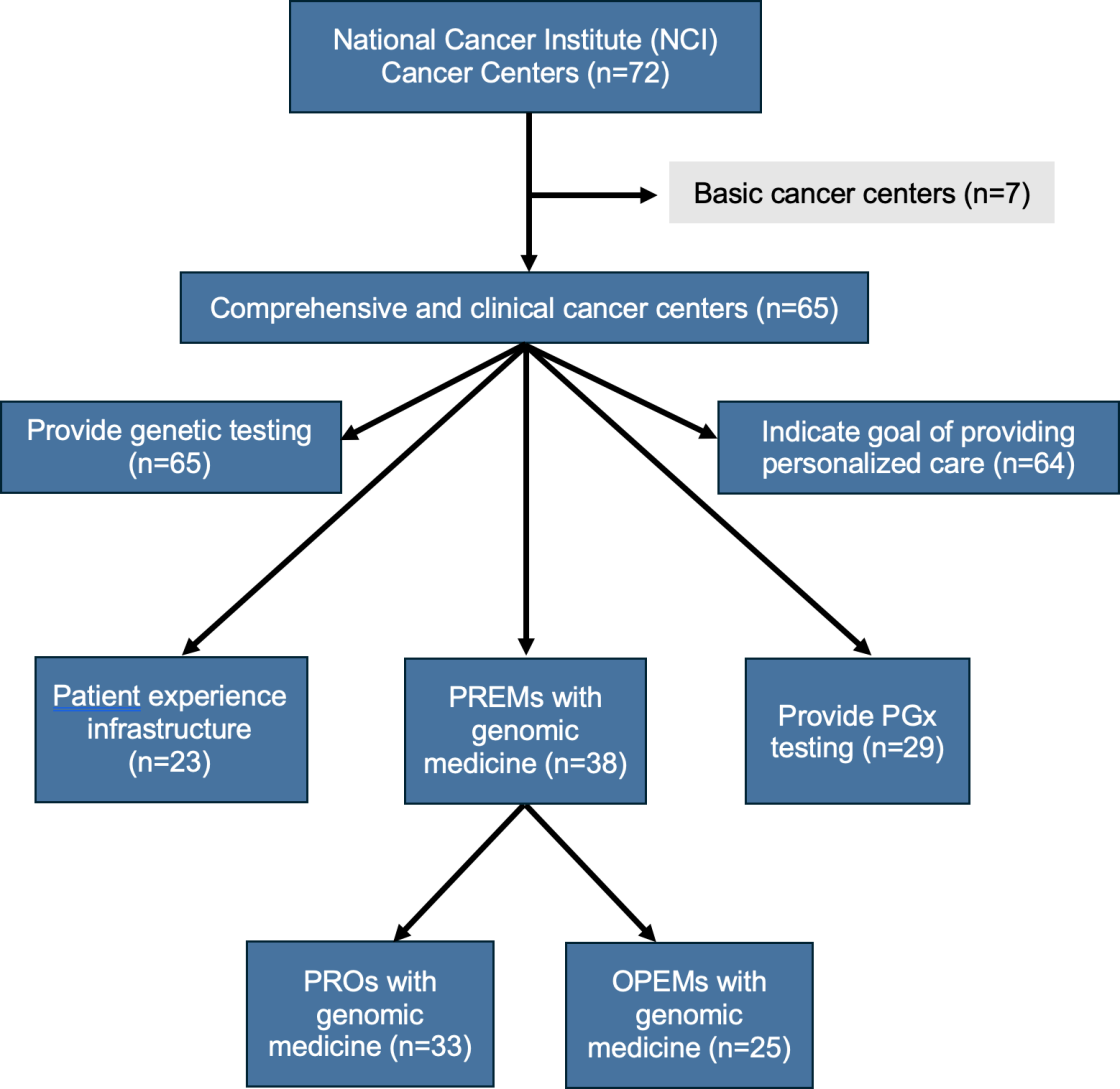
Flow diagram illustrating the criteria for the selection of cancer centers for content analysis and the domains on which National Cancer Institute–designated comprehensive cancer center websites were evaluated. OPEM: other patient experience measure; PGx: pharmacogenomics; PREM: patient-reported experience measure; PRO: patient-reported outcome.

Before the start of the evaluation of each center’s website, the 2 reviewers established definitions and criteria across five domains: whether NCI-CCs (1) provided genetic testing, (2) directly expressed a goal of delivering personalized care, (3) provided PGx testing, (4) assessed PREMs with genomic medicine (including PROs and OPEMs), and (5) indicated an established infrastructure or set of resources to evaluate patient experience (Figure 1). For each domain, we determined a set of phrases and terms to search for within each cancer center site to compile relevant results, which we further evaluated for specific content. All percentages reported in the tables reflect the proportion of all NCI-CCs (N=65) observed to publicly communicate content regarding each domain on their websites.

To assess the first domain of whether genetic testing of any kind was mentioned or provided by the cancer center, we searched for generic terms including “genetic test,” “genetic counseling,” and “genomic medicine.” To mark each center as “yes” with respect to providing genetic testing, there had to be at least one instance in which genetic testing was made available to patients within their catchment area (eg, germline genetic testing was provided for hereditary cancer risk screening). Furthermore, with regard to the second domain of whether they described a goal of delivering personalized medicine, the relevant search terms included “precision medicine” and “personalized medicine” along with broader terms such as “personalized” and “individualized.” To classify centers as expressing this goal (and mark them as “yes”), they had to use the aforementioned terms in the context of personalized care being a center-wide mission and not be solely reflective of an individual provider or entity within the center.

For our third domain, we investigated whether centers publicly referenced PGx testing as a part of their research, clinical activities, or programs. We defined PGx broadly to ensure inclusion of germline genetic testing that is used to guide medication prescribing. In the cancer context, PGx testing can be used in reference to a range of biomarker testing to select the targeted cancer therapy most appropriate for a patient’s tumor type [45,46]. Through targeted search phrases such as “personalized medicine” and “pharmacogenomics,” we identified centers that described the use of PGx testing to optimize patient drug therapies, inclusive of chemotherapy regimens, adjuvant therapies, and other supportive medications for oncology patients. We did not wish to include only tumor-specific somatic mutations as a form of PGx in this domain. Although tumor profiling is more common in pathology analysis and has been widely adopted [13], the consistent use of germline PGx to identify hereditary risk, improve drug efficacy, or reduce treatment toxicity is still an emerging area of personalized medicine and is indicative of NCI-CCs’ work to incorporate new innovative genetic technologies [47].

For the fourth domain, the inclusion of PREMs with genomic medicine opportunities was evaluated. To be marked as “yes” for this domain, the centers had to demonstrate the incorporation of at least one PREM concurrent with the use of genetic testing in either clinical practice or a clinical trial context. Following our assessment of the presence of genomic programs in the first domain, we identified the presence of PREMs in coordination with these programs and efforts at the NCI-CCs as indicated through clinical study design, clinical outcome data, or published patient results that explicitly mentioned collection of patient feedback. We further differentiated within this domain by classifying PREMs as either PROs or OPEMs with genomic medicine. PROs included any direct patient-reported measures used or collected in clinical care (eg, quality of life or mood tracking). OPEMs included any type of patient feedback beyond that used in direct clinical care, such as the impact of genomic medicine on their life, preferences for information delivery, their understanding of results, or risk communication with their care team.

Incorporating consideration of patient experiences in cancer care can improve cancer care and the accessibility of new technologies such as genetic testing [21]. For the fifth and final domain, we evaluated whether NCI-CCs had a previously established built infrastructure or dedicated resources specific to incorporating patient experiences into cancer care delivery. The presence of such infrastructure was initially identified from reviewing the results obtained using search phrases including “patient experience” and “patient engagement” before content review. Resources identified that we considered as a “yes” could include an office or department dedicated specifically to patient experiences [48,49], systematic surveying of patient experiences, a patient experience board, and other center-wide efforts concerning patient experiences. We recognize that this fifth domain is inclusive of very different types of resources that may potentially create variation in their impacts on centers in terms of accountability.

To ensure the accuracy of our determinations, the primary reviewer checked each website for these domains in 2 separate rounds, and the second reviewer checked to ensure the accuracy of the determinations. Both reviewers met regularly to discuss and resolve any discrepancies within the determinations. All results for these determinations were stored in a database and evaluated for quality control. Figure 1 shows the raw results for the presence of resources and communication among these domains.

Finally, we stratified NCI-CCs based on whether they indicated the presence of patient experience infrastructure (fifth domain) to assess the impact of having this established set of resources on public communication about the other domains of personalized medicine and patient experience measures of interest described previously. We recognize that, as the first analysis of its kind related to genomic medicine, our approach for stratification may be broad, yet it establishes a foundation upon which future analyses can build to provide more granular characterizations of patient infrastructure resources to further evaluate their possible influence on cancer centers.

### Ethical Considerations

All data were aggregated and publicly available; thus, no ethics approval, exemption, or deidentification process was needed.

## Results

Content analysis revealed that all 65 NCI-CCs evaluated indicated that they provided genetic testing of some sort to guide cancer care, and all cancer centers except 1 (64/65, 98%) explicitly indicated a center-wide goal of providing personalized care to their patients (Figure 1). In some cases, NCI-CCs had dedicated genetic counseling departments or even online eligibility screeners for patients interested in genetic testing, whereas others solely described the availability of genetic testing to patients through a clinical trial. However, slightly less than half (29/65, 45%) of the NCI-CCs discussed PGx testing in some form within their core resources, molecular tumor boards, or research activities (Table 1).

**Table 1.** Number and percentage of National Cancer Institute–designated comprehensive cancer centers (NCI-CCs) that described activities related to genomic medicine (N=65).

Activities related to genomic medicine	NCI-CCs, n (%)
Describing personalized care	64 (98)
Assessing PROs^a^ with genomic medicine	33 (51)
Assessing OPEMs^b^ with genomic medicine	25 (38)
Providing PGx^c^ testing	29 (45)
Indicating patient experience infrastructure	23 (35)

a PRO: patient-reported outcome.

b OPEM: other patient experience measure.

c PGx: pharmacogenomics.

Overall, we found that most of the NCI-CC websites (38/65, 58%) indicated the use of PREMs with genomic medicine, whether through incorporating PROs in clinical care or evaluating OPEMs. Among NCI-CCs using PREMs in some capacity, assessing PROs with genomic medicine was the most common, with approximately half (33/65, 51%) of all centers indicating that they assessed factors such as quality of life or symptom monitoring for patients receiving genomic-guided cancer care (Table 1). OPEMs with genomic medicine, representing broader aspects of patient experience beyond direct clinical care, such as evaluations of patient education and communication feedback, were described by 38% (25/65) of the NCI-CCs (Table 1).

In our stratified evaluation of whether the NCI-CCs had a previously established patient experience infrastructure, we found that approximately 1 in 3 NCI-CCs (23/65, 35%) indicated on their websites having dedicated center resources for evaluating and incorporating patient experience (Table 2). Furthermore, our content analysis showed that the presence of an established patient experience infrastructure was directly related to increased rates of the other measures relevant to personalized medicine and PREMs with genomic medicine (Table 2). For example, 57% (13/23) of the cancer centers with patient experience infrastructure demonstrated the assessment of PROs with genomic medicine compared to 48% (20/42) of the centers that did not indicate the same dedicated resources. Centers that communicated having dedicated resources to incorporate patient experience were also 7% more likely to assess OPEMs (10/23, 43% with dedicated resources vs 15/42, 36% without). Moreover, over half (12/23, 52%) of the centers that demonstrated having an established infrastructure provided PGx testing, yet only 40% (17/42) of those without this infrastructure provided the same opportunity for PGx-guided care.

**Table 2.** Number and percentage of National Cancer Institute–designated comprehensive cancer centers (NCI-CCs) that communicated about specific activities related to genomic medicine stratified by the presence of an established infrastructure and dedicated resources for incorporating patient experience.

Activities related to genomic medicine	NCI-CCs without patient experience infrastructure (n=42), n (%)	NCI-CCs with patient experience infrastructure (n=23), n (%)
Assessing PROs^a^ with genomic medicine	20 (48)	13 (57)
Assessing OPEMs^b^ with genomic medicine	15 (36)	10 (43)
Providing PGx^c^ testing	17 (40)	12 (52)

a PRO: patient-reported outcome.

b OPEM: other patient experience measure.

c PGx: pharmacogenomics.

## Discussion

### Principal Findings

In this study, we evaluated the publicly available online content presented by NCI-CCs communicating their efforts to incorporate patient experience with genomic medicine. Our content analysis revealed that all the NCI-CCs discussed incorporating genetic testing in some form or another. In addition, we found a high prevalence of statements emphasizing commitment to personalized care throughout the NCI-CCs’ websites. Our content analysis revealed variation between NCI-CCs in the type and content of information publicly communicated relevant to such personalized cancer care, particularly as it related to the incorporation of patient experience measures into cancer center programs. Finally, we discovered that cancer centers that had invested in building an established infrastructure focused on monitoring patient experience were more likely to provide information and describe the breadth of their activities to enhance patient-centered care.

Given the importance of genetic testing to providing personalized cancer care, we expected to see prominent levels of cancer center communication regarding these mission-driven priority areas. In spite of an expressed commitment to these areas of care, only about half of the nation’s cancer centers indicated the incorporation of patient experience measures with genomic medicine. Even fewer centers communicated about their evaluation of patient experiences with receiving genetic testing beyond the PROs typically accompanying clinical care. Less attention to OPEMs created gaps in knowledge related to patient views, preferences, and values regarding the use of genetics to guide cancer care across the continuum. Both PROs and OPEMs illuminate aspects of the patient experience that could contribute to disparities in cancer health outcomes [14,15]. Comprehensive integration of the patient perspective improves quality of care and facilitates centers’ efforts to accomplish their goal of providing patient-centered care [27]. Understanding and incorporating the patient experience into health care delivery is an established method of improving appropriately tailored implementation of genetic testing into the care received by a diverse patient population and their families [21,23,50].

Our stratified evaluation showed that those centers with a previously established infrastructure for integrating patient experience were more likely to communicate targeted activities to assess patient experience with genomic medicine. On the basis of our findings, the NCI-CCs that made investments in a patient experience infrastructure, such as an office dedicated to patient experience or patient advisory boards, communicated a wider range of activities to leverage patient experience and promote more equitable access to genomic medicine. NCI-CCs with such an established infrastructure were also more likely to communicate about the use of PGx testing to guide medication selection. Concerted efforts by NCI-CCs to disseminate information about the incorporation of patient experience may be one critical step toward augmenting public knowledge about the use of genetics in cancer care. As implementation and widespread use of these emerging genomic technologies increases, establishing such resources to amplify the patient voice may help enhance the accessibility of information to underserved and underrepresented populations in these NCI-CC catchment areas [51], for example, serving as a key conduit regarding eligibility for clinical trials incorporating genetic susceptibility, which often lack diversity [52-54].

The NCI emphasizes the long-term commitment to community outreach and engagement (COE) that is required to have a profound impact on the cancer burden of a center’s catchment area, and consequently, their primary metric in evaluating the strength of COE is the scope, quality, and impact of the center’s COE activities on the burden of cancer in the center’s stated catchment area [2]. Our study results suggest ample opportunities for cancer centers to leverage their websites to foster a more bidirectional sharing of information between cancer centers and the patients of their catchment areas. Key recommendations for centers to improve patient engagement and communication focus on building on the foundations of each center’s COE efforts, which are strategically tailored for their catchment area. The evolution of COE has enhanced its integration throughout cancer center programs and leadership activities [55]. While some NCI-CCs may be well-resourced enough to establish separate offices or departments dedicated to patient experience, others may augment collaborations with COE on public communication about patients’ experiences with personalized cancer care, whether through online sources such as the website or through the multilevel partnerships that promote innovation that cancer centers’ COE efforts are uniquely positioned to develop. In addition, even well-resourced NCI-CCs could improve the efficiency of their investments in incorporating patients’ experiences with personalized cancer care through engagement with COE.

This study has some limitations. Our evaluations of the NCI-CCs’ resources and communication on these issues were limited to their publicly available websites and did not account for other forms of communication including news articles, direct patient-provider communications, or materials distributed in person to patients. Furthermore, NCI-CCs’ varying resources may impact whether they have a dedicated infrastructure for enhanced communications or for enhancing patients’ experiences. Centers with comparatively more resources to support such a dedicated infrastructure may have a greater ability to allocate more resources to communicating effectively with patients (eg, public relations teams and website support services). Previous research has shown that, in NCI-CCs across the United States, catchment areas are extremely diverse in size, composition, cancer incidence, and mortality rates [56]. Catchment areas reflect the geographic area where centers focus their research, outreach, and engagement efforts to reduce cancer burden. However, despite these differences across the populations served, Cancer Center Support Grant funding varies widely across these centers serving catchment areas with varying intensity of needs. Thus, resource inequity may impact website quality due to having to choose where to allocate limited resources. A center with comparatively fewer resources might need to direct them toward sustaining offerings versus enhancing their website. In that case, a center’s website may be misleading as to the full scope of their actual offerings to enhance patient care. Nevertheless, NCI-CCs’ websites often serve as the first and most prominent public representation of a center to interested patients, reflecting their ongoing efforts and directly communicating center priorities.

Second, this study’s approach as a content analysis of cancer center websites did not confirm (1) efforts or activities to incorporate patient experience measurement into cancer center activities, (2) genetic data collection, or (3) the implementation of genetics into clinical care. However, outside of the patient-provider interaction, public information on cancer center websites serves as a valuable resource for patients in cancer center catchment areas to become informed about their cancer care and the resources available to them that they can pursue or advocate for [37]. In these cases, online information is crucial to expanding communication with patients and opportunities for health education beyond the limited time frame of the health encounter. Cancer center websites can bolster communication efforts with the community, provide information transparency, and promote resources to patients [38]. Future work will assess in more detail the variation in implementation strategies that cancer centers pursue regarding incorporating the patient perspective into personalized cancer care.

### Conclusions

As leaders in cancer care, NCI-CCs are poised to promote accessibility of information about advancements in genomic medicine being implemented into clinical care. Our findings show that, while genetic testing may be available, many NCI-CCs do not indicate currently monitoring patients’ experiences with genomically guided cancer care. It is particularly important to determine how many NCI-CCs report providing personalized care and assessing PROs via their public websites to provide a greater understanding of the scope of future opportunities for improved communication with patients in their catchment areas about patient-centered care within federally-funded centers with responsibilities for cancer care delivery innovation. To our knowledge, this is the first study to evaluate cancer centers’ communication with the public about incorporating patient experience, engagement, and communication regarding genomic medicine. Moreover, to our knowledge, the NCI neither publishes for public consumption nor provides any explicit guidelines specifically tailored for genomic medicine. Lack of public communication on a critical tool for personalizing care presents a key area for improvement in the communication efforts of NCI-CCs. Systematic efforts to incorporate patient experience could serve as one mechanism for cancer center outreach to the diverse patient populations they serve, as well as act as another step toward achieving their stated goals of providing high-quality, personalized cancer care.

## Abbreviations

COE: community outreach and engagement
NCI: National Cancer Institute
NCI-CC: National Cancer Institute–designated comprehensive cancer center
OPEM: other patient experience measure
PGx: pharmacogenomics
PREM: patient-reported experience measure
PRO: patient-reported outcome
